# Disinhibition of Primitive Reflexes in Attention Deficit and Hyperactivity Disorder: Insight Into Specific Mechanisms in Girls and Boys

**DOI:** 10.3389/fpsyt.2021.430685

**Published:** 2021-11-08

**Authors:** Petr Bob, Jana Konicarova, Jiri Raboch

**Affiliations:** ^1^Center for Neuropsychiatric Research of Traumatic Stress, Department of Psychiatry and UHSL, First Faculty of Medicine and Department of Psychiatry, Faculty of Medicine in Pilsen, Charles University, Prague, Czechia; ^2^Stroder Therapy Center, Cham, Germany

**Keywords:** ADHD (Attention Deficit and Hyperactivity Disorder), Asymmetric Tonic Neck Reflex, balance deficits, developmental disorders, dissolution, primitive reflexes integration, Symmetric Tonic Neck Reflex

## Abstract

**Objective:** Cognitive and motor disintegration and other functional disturbances in various neuropsychiatric disorders may be related to inhibitory deficits that may manifest as a persistence or re-expression of primitive reflexes and few recent data suggest that these deficits may occur in Attention Deficit and Hyperactivity Disorder (ADHD).

**Methods:** We have tested a hypothesis to which extent ADHD symptoms and balance deficits are related to persisting primitive reflexes, such as Asymmetric Tonic Neck Reflex (ATNR) and Symmetric Tonic Neck Reflex (STNR) in 80 medication-naïve children with ADHD (40 boys and 40 girls) in the school age (8–11 years) and compared these data with a control group of 60 children (30 boys and 30 girls).

**Results:** These data show new finding that ADHD symptoms and balance deficits are strongly and specifically associated with persistent ATNR in girls and STNR in boys.

**Conclusions:** These results provide first evidence in medical literature that ADHD in girls and boys is specifically related to distinguished neurological developmental mechanisms related to disinhibition of primitive reflexes.

## Introduction

According to recent findings functional disturbances due to various insults such as brain damages, toxic influences or psychological stress may manifest in various stages of development and an early brain insult or trauma may influence pathological changes that may manifest later in life (1, 2). A specific developmental disorder is Attention Deficit and Hyperactivity Disorder (ADHD) which is closely associated with cognitive and motor disintegration, inhibitory dysfunctions, balance deficits and other functional disturbances developed in early stages of life (3, 4). These ADHD developmental deficits may be related to persistence or re-expression of the primitive reflexes, as for example Symmetric Tonic Neck Reflex (STNR) and Asymmetric Tonic Neck Reflex (ATNR) (5). According to current findings there is no evidence about relationships of ADHD symptoms to manifestations of the primitive reflexes and balance deficits that have been found in several neuropsychiatric disorders (6).

These developmental disturbances may be understood within the concept of “dissolution” proposed by Hughlings Jackson, who proposed that later developed functions during ontogenesis of the central nervous system (CNS) tend to replace the older ones (7, 8). This concept implicates that when higher stages of the CNS development were not successfully achieved or were damaged, the lower and more primitive neural functions may be disinhibited and their release from control can lead to dysregulation of later developed brain functions (7). This general concept of developmental neurology seems to be in agreement with evidence of hypofrontality in ADHD (9, 10), which suggests that deficits of higher attentional and executive functions are related to reduced activations in the right inferior frontal cortex, anterior cingulate cortex, supplementary motor area and other regions (11).

To the best of our knowledge there is no evidence in the scientific literature about relationships of ADHD symptoms, persistence (or re-expression) of the primitive reflexes and balance deficits. With respect to these findings we have tested a hypothesis to which extent ADHD symptoms are related to persisting primitive reflexes ATNR and STNR, and balance deficits in 80 medication-naive children with ADHD (40 girls and 40 boys) in the school age (8–11 years). For comparison of these data we also assessed a control group of 60 children (30 boys and 30 girls).

## Methods

### Participants

In collaboration of the Center for Neuropsychiatric Research of Traumatic Stress and the ELSPAC study (European Longitudinal Study of Parenthood and Childhood) data of 80 medication-naive children, including 40 girls (mean age 9.21, SD = 1.14 age range 8–11) and 40 boys (mean age 9.30, SD = 1.11 age range 8–11), with ADHD diagnosed according to DSM-IV criteria were collected. The collected data included scores of primitive reflexes ATNR and STNR, balance deficits measured as soft neurological signs and psychometric measure of ADHD symptoms. All the patients were medication-naïve and their assessments were done before starting treatment. The ADHD diagnosis was combined hyperactive-inattentive subtype with occurrence of ADHD based learning difficulties. Exclusion criteria were other child psychiatric and neurological disorders, obesity and metabolic disorders, substance abuse and also previous exposure to stimulant medication, left-handedness and intelligence lower than average according to Wechsler Intelligence Scale for Children (WISC-IV). Recruitment was based on consecutive visits of the untreated patients in the clinic (Psychosomatic Clinic, Waldmunchen and Stroder Therapy Center, Cham, Germany) and the control group was recruited through school advertising in the same cities. The control group of 30 girls (mean age 9.13, SD = 1.13, age range 8–11) and 30 boys (mean age 9.33, SD = 1.18, age range 8–11) without ADHD fulfilled the same exclusion criteria. Parents of all the participants gave written informed consent and the research was approved by Charles University, First Faculty of Medicine Ethical committee in collaboration of the project ELSPAC and Charles University research project of the Center for Neuropsychiatric Research of Traumatic Stress.

### Measurement of Balance Deficits

For assessment of balance deficits we have used four items from the Physical and Neurological Examination for Soft Signs scale (PANESS) by Denckla (12) that can be used in children and adolescents. PANESS is an observational scale with 21 tasks which includes also the items useful for measurement of balance deficits:

Standing eyes closed- Item 9 “Sustentation Postures/Stations- “Now I want you to put one foot in front of the other, just as you did for walking the straight line forward and backwards right now, and close your eyes. Stay that way as long as you can, or until I tell you to relax.” Time with stopwatch duration of success up to 20 sec. Score: Use stopwatch-determine time to score in groupings (see form) of duration. Code for tendency to fall (1) and for bringing arms up and out to balance (1). Scoring is on the scale 0, 1, 2, 3.

Walking Forward-Item 7 Tandem Walking: “Now be sure you put your heel against your toe and walk to the end staying on the line.” Score: An error consists of not placing the heel to toe or missing the line completely. Code number of gaps, misses, failure to place heel to toe. Scoring is on the scale 0, 1, 2, 3.

Walking Backward*-*Item 8 Tandem Walking: “Now do the same thing backwards.” Score as in test for forward tandem gait on the scale 0, 1, 2, 3.

Walking Forward*-*Item 7 Tandem Walking- the same procedure and scoring with eyes closed. Score on the scale 0, 1, 2, 3. The measurements of balancing deficits was done twice by two independent examinators and averaged.

### Measurement of Primitive Reflexes

Asymmetric tonic neck reflex (ATNR) usually measured by Schilder Test (13) presents the tonic reflex response that occurs in newborn babies and normally vanishes around 3 months of age. Test position is standing, feet together, with the arms held straight out at shoulder level and height, but with the hands relaxed at wrists. In the test procedure the tester stands behind the subject and gives the instruction: “When I turn your head. I want you keep your arms straight out in front of you, as they are now. This means your arms remain in the same position, and only your head moves.” Then the tester slowly rotates the subject's head until the chin is parallel with the shoulder. Pause for 10 s. Return the head to the midline. Pause for 10 s. Rotate the head to the other side and pause for 10 s. Repeat the procedure up to 4 times. Typical indicators of this reflex included movement of the extended arms in the same direction as the head turn, dropping of the arms or waying and loss of balance. Scoring: 0- no response which means the arms remain straight out in front; 1-slight movement of the arms up to 20° to the same side as the head is turned or slight dropping of the arms; 2-movement of the arms up to 45° as the head is turned or marked dropping of the arms; 3-arm movement >45° either to the side or down, swaying or loss of balance.

Symmetric tonic neck reflex measured by Bender-Purdue Reflex Test (14) merges 6–8 months of life and is inhibited between 9 and 11 months. The subject is instructed to maintain the test position but to slowly move the head to look down “as if looking between your thighs.” Hold the position for up to 5 s and then slowly move the head upwards “as if looking at the ceiling” Repeat up to 6 times. Typical indicators of the reflex are any bending of the arms as a result of head flexion and/or raising the feet. Straightening of the arms and flexion of the knees as a result of head extension. Any bending of the arms as a result of head flexion and/or raising of the feet. Straightening of the arms and flexion of the knees as a result of head extension. Scoring: 0 - no response; 1 - tremor in one or both arms or slight hip movement; 2 - movement of the elbow on either side and/or definite, movement in the hips, or arching of the back; 3 - bending of the arms on head flexion or movement of the bottom back on head extension or bending of the arms to the floor, or movement of the bottom back onto the ankles, so that the subject is sitting in the cat position.

The measurements of the primitive reflexes were done twice by two independent examinators and averaged.

### Psychometric Measurement of ADHD Symptoms

Frequently used measure of ADHD symptoms is Conners' Parent Questionnaire (CPQ) (15). The CPQ is 93-item scale of symptoms that are most commonly associated with behavioral disorders and related to children and adolescents (aged 3–17), and can measure treatment changes and outcome assessment purposes. In this study a validated German version was used which has equivalent psychometric properties as the English original (16).

The questionnaire enables to calculate total score and it has also subscales based on factor structure of the questionnaire—I. Conduct problems (items 39, 40, 41, 47, 48, 51, 69); II. Anxiety (items 8, 9, 10, 11, 42, 43, 64); III. Impulsivity-hyperactivity (items 78, 80, 81, 82, 83, 84, 89, 90); IV. Learning problems (items 45, 62, 63, 67); V. Psychosomatic difficulties (items 6, 21, 22, 23, 24); VI. Perfectionism (items 3, 76, 77, 78); VII. Antisocial behavior (items 71, 72, 73, 75); VIII. Muscular tension (items 12, 13, 14, 36). The symptoms are rated on a 4-point Likert scale (from 0 to 3) by either one or both parents of the child. In this study CPQ was completed by child's mother and the responses were communicated with a psychologist performing the examination to assess their validity.

### Statistical Analysis

Statistical evaluation of scores of the measures of ATNR, STNR, balance deficits and ADHD symptoms included descriptive statistics, Kruskal-Wallis ANOVA and Spearman correlation coefficients. To prevent Type II error which would disable to reject null hypothesis that ADHD symptoms are not linked to balancing deficits and primitive reflexes we performed Power Analysis and assessed the effect sizes characterizing correlation coefficients. The statistical evaluations were performed using the software package Statistical Version 6.

## Results

The main results of this data analysis show that ADHD symptoms measured by total score of the Conners' Parent Symptom Questionnaire (*CPQ*) are significantly correlated with scores of persisting primitive reflexes *ATNR* in girls (Spearman *r* = 0.55, *p* < 0.01) and with scores of persisting primitive reflexes *STNR* in boys (Spearman *r* = 0.52, *p* < 0.01).

Other results indicate that CPQ total score is also significantly correlated with scores of balance deficits during *Standing with eyes closed* (girls Spearman *r* = 0.42, *p* < 0.01; boys Spearman *r* = 0.40, *p* < 0.01), *walking forward* (girls Spearman *r* = 0.37, *p* < 0.05; boys Spearman *r* = 0.39, *p* < 0.05) and *walking forward with closed eyes* just in boys (Spearman *r* = 0.50, *p* < 0.01). Other correlations of CPQ subscales with primitive reflexes and balance deficits are summarized in Table 1. These correlations show that ADHD symptoms are specifically associated with persisting ATNR in girls and STNR responses in boys.

**Table 1:** Spearman correlations of balancing scores with primitive reflexes and CPQ total score and its subscales in 40 girls and 40 boys with ADHD.

	**Standing eyes closed**		**Walking fwd**		**Walking back**		**Walking fwd eyes closed**		**ATNR reflex**		**STNR reflex**	
	**Girls**	**Boys**	**Girls**	**Boys**	**Girls**	**Boys**	**Girls**	**Boys**	**Girls**	**Boys**	**Girls**	**Boys**
ATNR reflex	**0.43**	0.02	0.06	0.00	−0.04	0.16	0.14	0.16	1.00	1.00	**0.38**	0.21
STNR reflex	−0.07	0.05	0.14	−0.01	0.07	−0.05	0.22	**0.52**	**0.38**	0.21	1.00	1.00
CPQ	**0.42**	**0.40**	**0.37**	**0.39**	−0.14	−0.01	0.14	**0.50**	**0.55**	0.17	0.23	**0.50**
I. Conduct probl.	0.09	**0.46**	0.15	0.28	−0.28	−0.08	0.01	**0.40**	0.21	0.09	0.10	**0.34**
II. Anxiety	**0.48**	0.01	0.20	0.29	−0.08	0.01	0.09	0.03	**0.32**	0.13	−0.08	0.00
III. Impuls-hyperact.	**0.34**	0.29	**0.39**	0.16	0.01	0.01	0.17	0.22	**0.38**	**0.36**	0.26	**0.38**
IV. Learning problems	−0.11	0.04	**0.41**	0.25	−0.20	−0.06	−0.19	0.13	0.24	−0.03	**0.40**	**0.37**
V. Psychosomatic	0.04	0.10	0.21	0.09	−0.12	0.19	−0.01	**0.32**	0.07	0.05	0.01	0.10
VI. Perfectionism	0.24	−0.25	0.26	0.06	**0.31**	0.23	**0.41**	−0.13	0.28	**0.37**	0.08	0.02
VII. Antisocial behav.	0.045	0.20	−0.25	0.25	−0.28	0.15	−0.15	0.04	0.16	**0.33**	−0.17	0.21
VIII. Muscular tension	0.08	0.17	0.04	0.19	−0.23	0.13	−0.04	**0.47**	0.25	0.23	−0.04	**0.38**

*Spearman correlation coefficients higher than 0.40 are significant at p < 0.01, with the Fisher z > 0.43 and correlation coefficients higher than 0.31 are significant at p < 0.05, with the Fisher z > 0.32. Bold correlations are statistically significant*.

Other results also show that scores of balance deficits during *Standing with eyes closed* are significantly correlated with scores of persisting primitive reflexes *ATNR* in girls (Spearman *r* = 0.43, *p* < 0.01) and balance deficits during *Walking fwd with eyes closed* are significantly correlated with scores of persisting primitive reflexes *STNR* in boys (Spearman *r* = 0.52, *p* < 0.01). Other correlations of balance deficits with scores of primitive reflexes were not statistically significant (Table 1).

For comparison of the results we have used data from the control group of 60 children (30 girls and 30 boys) of the same age, who were assessed for CPQ, ATNR, and STNR scores and balance deficits. The control group of children was without ADHD symptoms. From the control group 2 boys manifested mild ATNR response and 1 girl and 2 boys manifested STNR response (in all these cases with the score 1). Other children did not manifest ATNR or STNR response. The control group also manifested very rare and minor balance deficits that on the scale from 0 to 3 had maximum value 1, which occurred in 3 girls and 4 boys in standing with eyes closed, in 1 boy during walking forward, in 1 girl and 1 boy during walking back, and in 3 girls and 6 boys during walking back with eyes closed. Other children did not manifest balance deficits. The groups of girls and boys in statistical analysis using Kruskal-Wallis ANOVA were not statistically different with respect to age and intelligence.

## Discussion

Most significant results of this study show that levels of ADHD symptoms and balance deficits in girls are associated with persisting ATNR, whether ADHD symptoms and balance deficits in boys are closely related to persisting STNR. These data indicate that ADHD in boys and girls are linked to different neurological developmental pathways that are related to specific disinhibition and persistence of these primitive reflexes, ATNR in girls and STNR in boys.

In agreement with these results recent findings show that high proportion of children with ADHD manifest altered balance and motor abnormalities (3, 4). According to brain imaging studies balance deficits likely are linked to prefrontal cortex dysfunctions that influence attention and executive functions (17–19). These deficits also may be related to cerebellar disinhibition in agreement with findings that ADHD children in many cases have abnormalities in cerebellar regions associated with balance and gait control (3, 20, 21).

The novel findings of this study show that cognitive and motor dysfunctions, and disturbed balance are strongly associated with persisting primitive reflexes (ATNR in girls and STNR in boys) that were not sufficiently suppressed and inhibited in later stages of development. According to recent evidence persistence or re-expression of primitive reflexes may occur in various neuropsychiatric syndromes such as schizophrenia, various forms of dementia (5, 6, 22, 23) and were reported also in patients with dyslexia (24, 25), and in school aged children with ADHD (26–29).

In summary, results of this study show that ADHD symptoms and balance deficits are gender-specifically related to the persisting primitive reflexes ATNR and STNR. This relationship to the best of our knowledge represents the first documented finding that ADHD development in boys and girls is related to neurologically different developmental pathways that specifically influence disinhibition of ATNR girls and STNR in boys. Together these data indicate that ADHD symptoms may represent the process related to “neural dissolution” and “interference” of more primitive neural mechanisms, such as primitive reflexes ATNR and STNR, with higher levels of brain functions due to insufficiently developed cognitive and motor integration. In this context, gender differences in these manifestations might be related to specific and delayed brain maturation in boys compared to girls (30).

These documented neurological differences related to gender-specific ADHD neurodevelopmental mechanisms need further research that may enable to develop new gender-specific neurorehabilitation methods for ADHD therapy focused on integration of primitive reflexes, as for example application of movement intervention programs (13, 24). These methods might be useful as therapeutic interventions in various conditions in the clinic and also as everyday training in schools. According to the preliminary evidence reported by McPhillips et al. (24), the specific movement program focused on repetition of stereotypical neonatal movements related to patterns of primitive reflexes seems to be very useful treatment method. In this follow-up study McPhillips et al. documented decreased ATNR manifestations and improving educational skills in children with dyslexia in comparison to placebo and control groups. These therapeutic interventions and their prospective development may be very important with respect to specificity of ATNR manifestations in girls, and STNR in boys which indicate gender-specific neural mechanisms of ADHD.

## Data Availability Statement

All datasets generated for this study are included in the article and/or the supplementary files.

## Ethics Statement

The study was approved by Charles University Ethical Board.

## Author Contributions

PB, JK, and JR: collecting data, data analysis, and writing the manuscript. All authors contributed to the article and approved the submitted version.

## Funding

The study was supported by projects Progress provided by Charles University.

## Conflict of Interest

The authors declare that the research was conducted in the absence of any commercial or financial relationships that could be construed as a potential conflict of interest.

## Publisher's Note

All claims expressed in this article are solely those of the authors and do not necessarily represent those of their affiliated organizations, or those of the publisher, the editors and the reviewers. Any product that may be evaluated in this article, or claim that may be made by its manufacturer, is not guaranteed or endorsed by the publisher.
